# Feeding Behavior Modulates Biofilm-Mediated Transmission of *Yersinia pestis* by the Cat Flea, *Ctenocephalides felis*

**DOI:** 10.1371/journal.pntd.0004413

**Published:** 2016-02-01

**Authors:** David M. Bland, B. Joseph Hinnebusch

**Affiliations:** Laboratory of Zoonotic Pathogens, Rocky Mountain Laboratories, National Institute of Allergy and Infectious Diseases, National Institutes of Health, Hamilton, Montana, United States of America; University of California San Diego School of Medicine, UNITED STATES

## Abstract

**Background:**

The cat flea, *Ctenocephalides felis*, is prevalent worldwide, will parasitize animal reservoirs of plague, and is associated with human habitations in known plague foci. Despite its pervasiveness, limited information is available about the cat flea’s competence as a vector for *Yersinia pestis*. It is generally considered to be a poor vector, based on studies examining early-phase transmission during the first week after infection, but transmission potential by the biofilm-dependent proventricular-blocking mechanism has never been systematically evaluated. In this study, we assessed the vector competence of cat fleas by both mechanisms. Because the feeding behavior of cat fleas differs markedly from important rat flea vectors, we also examined the influence of feeding behavior on transmission dynamics.

**Methodology/Principal Findings:**

Groups of cat fleas were infected with *Y*. *pestis* and subsequently provided access to sterile blood meals twice-weekly, 5 times per week, or daily for 4 weeks and monitored for infection, the development of proventricular biofilm and blockage, mortality, and the ability to transmit. In cat fleas allowed prolonged, daily access to blood meals, mimicking their natural feeding behavior, *Y*. *pestis* did not efficiently colonize the digestive tract and could only be transmitted during the first week after infection. In contrast, cat fleas that were fed intermittently, mimicking the feeding behavior of the efficient vector *Xenopsylla cheopis*, could become blocked and regularly transmitted *Y*. *pestis* for 3–4 weeks by the biofilm-mediated mechanism, but early-phase transmission was not detected.

**Conclusions:**

The normal feeding behavior of *C*. *felis*, more than an intrinsic resistance to infection or blockage by *Y*. *pestis*, limits its vector competence. Rapid turnover of midgut contents results in bacterial clearance and disruption of biofilm accumulation in the proventriculus. Anatomical features of the cat flea foregut may also restrict transmission by both early-phase and proventricular biofilm-dependent mechanisms.

## Author Summary

Although plague primarily affects wild rodents, human plague outbreaks still occur periodically in some areas of the world. *Yersinia pestis*, the bacterial agent of plague, is transmitted by fleas. Cat fleas are abundant worldwide, regularly infest human habitations in plague endemic areas, and readily bite humans. The cat flea has long been considered to be a poor vector, but this reputation is based on very limited studies; and in general the reasons why some flea species are better vectors than others are not well understood. We found that cat fleas can transmit *Y*. *pestis*, but at rates strongly influenced by feeding frequency after the infectious blood meal. When infected cat fleas had unrestricted access to blood (their preferred feeding pattern), most rapidly cleared the infection and they transmitted only during the first week after infection. However, when post-infection blood meals were limited to mimic patterns typical of efficient flea vectors, cat fleas regularly transmitted for one month after infection. Our results indicate that cat fleas in their natural environments have limited potential to develop transmissible infections. However, if their regular daily feeding habit is interrupted, they can be capable vectors.

## Introduction

The cat flea, *Ctenocephalides felis*, is a mammalian ectoparasite commonly found on domesticated animals, and, by association, humans [1]. *C*. *felis* is a known vector for the bacterial pathogens *Rickettsia felis*, *R*. *typhi*, and *Bartonella* spp. [2–4]. However, relatively limited information is available regarding the cat flea’s competency as a vector for *Y*. *pestis* and its potential role in plague ecology and epidemiology. Cat fleas parasitize a wide range of animals including cats, dogs, livestock, wild mammals, and occasionally rats [1,5–8]. *C*. *felis* confirmed positive for *Y*. *pestis* have been collected from the Democratic Republic of Congo [9]. In addition, *C*. *felis* is the predominant species associated with human habitations in plague endemic regions of China and Uganda [10,11]. Their broad geographic range, low host specificity, and willingness to bite humans elevates the importance of investigating cat fleas as a vector of *Y*. *pestis* [12,13].

*Y*. *pestis* is the etiologic agent of plague, a zoonotic disease that is typically maintained in wild rodent populations. The most common presentation of the disease is bubonic plague, in which bacteria deposited into the dermis of a mammalian host from the bite of an infected flea migrate to the draining lymph node and quickly multiply. Without therapeutic intervention, plague bacilli frequently enter the host blood stream and disseminate throughout the body [14]. When a host is in the bacteremic stage of disease, fleas ingesting a blood meal can become colonized by *Y*. *pestis*. If the flea is unable to clear the infection, *Y*. *pestis* will produce an exopolysaccharide that allows bacteria to aggregate and form a biofilm in the flea digestive tract [15]. *Y*. *pestis* biofilms can form in the flea midgut, the proventriculus (a valve in the foregut that when closed prevents the reflux of ingested blood back into the esophagus), or in both locations [16,17]. As it gradually accumulates, the biofilm can completely block the passage of blood through the proventriculus, rendering the flea incapable of taking future blood meals [18]. Blocked fleas will make repeated feeding attempts, during which plague bacilli can be dissociated from the biofilm and refluxed into a feeding site [19].

Biofilm-mediated transmission can occur as early as one week after infection, but is more commonly observed after two weeks or more of infection [20]. This is a biological transmission mechanism, in that the bacteria actively colonize, replicate, and undergo developmental changes within the vector in order to become infectious [21–23]. The rat flea *Xenopsylla cheopis* is considered the most efficient vector of *Y*. *pestis* due to its high potential to become infected and blocked [24]. Reported blockage rates vary considerably among other flea species thought to be important vectors for *Y*. *pestis* [25]. However, most of these blockage rates were not determined in controlled laboratory settings with defined infectious doses, making comparisons between studies difficult to interpret.

*Y*. *pestis* can also be transmitted via a biofilm-independent mechanism termed early-phase transmission (EPT) [26–29]. This mode of transmission occurs primarily upon the first feeding of the flea within 4 days after an infectious blood meal containing a high titer of *Y*. *pestis* [30]. The mechanism of EPT has not been determined. The brief time period immediately after flea infection during which EPT occurs fits a mechanical transmission model whereby contamination of flea mouthparts with plague bacilli results in bacterial deposition into the host dermis [31]. However, regurgitation of digestive tract contents has been hypothesized to be a more likely explanation [30].

Previous studies of *C*. *felis* vector competence for *Y*. *pestis* primarily examined EPT using low numbers of infected fleas, short infection periods, and small animal models. The Indian Plague Research Commission [31] and Devignat [32] were able to successfully infect cat fleas with *Y*. *pestis*, but did not observe transmission when groups of 20–25 infected cat fleas were used to challenge naïve mice or guinea pigs during the first week after infection. These results are contrary to those of Verjbitski and Eisen *et al*., who did detect EPT by cat fleas, although at low efficiency compared to other flea vectors (5–33% transmission rate to mice or rats challenged by groups of 7–11 infected cat fleas) [26,33]. Wheeler & Douglas showed that 50 infected cat fleas placed on a naïve guinea pig during the first week after infection transmitted *Y*. *pestis*, but challenge with individual fleas did not result in transmission [27]. They also reported seeing evidence of infection but not proventricular blockage in histological sections of infected cat fleas [27,34]. More recently, Tam *et al*. demonstrated that *Y*. *pestis* could form a biofilm in the proventriculus of *C*. *felis*, indicating the potential for cat fleas to transmit through a biofilm-mediated mechanism [35]. Taken together, these studies suggest that cat fleas are capable yet inefficient at EPT but have not been evaluated for their ability to transmit via blockage-dependent transmission. The reason for the cat flea’s relative inefficiency as a vector by EPT has not been established.

A number of factors have been postulated to contribute to flea vector competence for plague including the anatomy of the flea proventriculus, physiological conditions in the gut, as well as environmental conditions such as temperature [17,20,36]. However, little is known about how different feeding behaviors influence vector competence. In the wild, cat fleas prefer to remain associated with their host and are frequent blood feeders. Females can ingest 15 times their body weight in blood per day [37]. In contrast, *X*. *cheopis* and *Oropsylla montana*, flea vectors frequently implicated in plague transmission cycles, feed much more intermittently [24,30,38,39].

In this study, we used standardized, well-defined methodologies to determine the infection potential, the incidence of proventricular blockage, and vector competence of cat fleas with respect to *Y*. *pestis*. Cat fleas were maintained using different feeding schedules after infection to determine whether their specialized feeding behavior influences long-term infection and transmission potentials. In addition, we compared the foregut anatomy of *C*. *felis* and *X*. *cheopis* to identify traits that could potentially influence the reflux of *Y*. *pestis* from the digestive tract and transmission efficiency.

## Materials and Methods

### Flea colony maintenance

Cat fleas were obtained from El Labs (Soquel, California) and were reared in a climate-controlled incubator at 25°C and 75% relative humidity (RH). The bottom of the high-walled container housing the flea colony contained a layer of flea bedding (mixture of fine sawdust, powdered milk, powdered mouse chow, and dried blood) for larval development and pupation. The colony was fed 8 hours/day, Monday through Friday on sterile defibrinated sheep blood (Quad Five, Rygate, Montana) using the 5W1 membrane feeding system (Hemotek Ltd, Lancaster, England). Parafilm “M” was stretched over the blood meal reservoir to serve as a feeding membrane [40]. Sterile gauze was tightly fitted over the parafilm membrane to aid in flea attachment and feeding. The feeding unit was set to 37°C and suspended above the bedding layer in the flea colony. *X*. *cheopis* fleas were from established colonies at Rocky Mountain Laboratories maintained as previously described [41].

### Flea infection

Adult cat fleas were removed from the colony and starved for 5 days prior to infection. Fleas were infected with the *Y*. *pestis* strain KIM6+ (Pgm^+^, Lcr^-^) transformed with pAcGFP1 (amp^r^), a plasmid that encodes a constitutively expressed green fluorescent protein gene (Clontech, Mountain View, CA). A frozen stock of *Y*. *pestis* KIM6+ (pAcGFP1) was used to inoculate brain heart infusion broth (BHI) supplemented with 10 μg/ml hemin and 100 μg/ml carbenicillin. Following 24 hours of incubation without aeration at 28°C, the culture was diluted 1:100 in 100 ml of BHI supplemented with carbenicillin and then incubated for 16–18 hours at 37°C without aeration. A volume of culture estimated by optical density measurement to contain ~1.2x10^9^ CFU was centrifuged at 5000 rpm for 5 minutes and the pellet was resuspended in 1 ml of sterile phosphate buffered saline (PBS). The bacterial suspension was added to 5 ml of heparinized mouse blood (initial bacterial concentration ~2x10^8^ CFU/ml, a biologically relevant level of bacteremia that has been routinely used for transmission studies [25,30,42]) and this blood was added to the feeding apparatus that has been described previously [15]. Approximately 300 fleas were given the opportunity to feed for 4 hours, collected, immobilized by placing them on ice, arrayed on a chill table, and screened using a dissecting microscope for the presence of fresh red blood in the midgut. Fleas that did not take a blood meal were removed from the study. Low- and high-frequency experimental group fleas fed on the same infectious blood meal and were then segregated; moderate-frequency group fleas were infected separately. Rat fleas were infected identically as described above but were only allowed to feed for 1 hour on the infectious blood meal. The bacterial concentration in the infectious blood meal was determined by removing a sample immediately after adding bacteria to the blood and again after the 4-hour feeding period, and plating serial dilutions on blood agar plates (BA) supplemented with carbenicillin. Colonies were counted after incubation at 28°C for 48 hours.

### Maintenance of infected fleas

Three groups of cat fleas (approximately equal numbers of males and females) were housed in plastic capsules and kept at 21°C, 75% RH. For every experiment, one capsule of ~100 infected cat fleas was used to monitor blockage, mortality, and bacterial transmission; and a second capsule of ~150–200 infected fleas was used to determine infection rates, bacterial titers, and severity of proventricular infection. A third capsule of ~100 cat fleas that fed on sterile blood served as a matching uninfected control and was used to track mortality. All three capsules were given maintenance (uninfected) blood meals over a 28-day period according to one of the following schedules: twice a week on Tuesdays and Fridays for 4 hours (low frequency), 5 times a week, Monday through Friday for 4 hours (moderate frequency), or daily for 18–22 hours (high frequency). Maintenance feeding schedules for all 3 groups began 22 hours after cat fleas were initially infected. For each maintenance feed, capsules containing the cat fleas were secured to the bottom of a Hemotek feeder, set up as described above and incubated at ambient room temperature (21–22°C) and humidity. Cat fleas fed at high frequency remained exposed to the feeding unit except for the 2–6 hours between feedings. Blood fed to the high-frequency group was supplemented with 100 μg/ml carbenicillin to prevent overgrowth of bacterial contaminants; this was not necessary for the low- and high-frequency groups, which fed for only 4 hours. Rat fleas received maintenance blood meals twice a week as previously described [43].

### Flea blockage, mortality, and infection rate

Immediately following each maintenance feed, flea mortality was recorded and the fleas were screened microscopically for signs of proventricular blockage (the presence of fresh red blood in the esophagus and not in the midgut). Upon diagnosis of blockage, blocked fleas were removed from the group and placed in a separate capsule to monitor their survival and transmission independently from the larger experimental populations. Blocked flea mortality was included in group mortality rate calculations. Non-blocked fleas were returned to the original capsule.

Samples of 10–20 fleas (approximately equal numbers of males and females) were collected immediately after the infectious blood meal, and at weekly intervals post-infection, and stored at -80°C. Subsequently, fleas were surface sterilized with successive washes of 3% hydrogen peroxide, 95% ethanol, and sterile distilled H_2_O. Individual fleas were mechanically disrupted in 1ml of PBS using lysing matrix H beads and a bead beating apparatus (MP Biomedicals, Santa Ana, California). Dilutions of the triturated fleas were plated in BHI soft agar overlays, supplemented with carbenicillin and 10 μg/ml hemin to determine the infection rate and average CFU per infected flea.

### Mass transmission

Following maintenance feedings on sterile blood, fleas were removed, the blood meal reservoir was detached from the heating unit, and the blood was collected, avoiding potential contamination from the outer surface of the parafilm. The entire 2.4 ml of blood was distributively plated on BA plates with carbenicillin. The reservoir was washed once with 500 μl of sterile PBS to recover any residual blood, which was also plated. CFUs were counted after 2–3 days incubation at 28°C; colonies were confirmed to be GFP-positive.

### Transmission by individual blocked cat fleas

The day after a flea was diagnosed as blocked, it was allowed to feed by itself for 4 hours in a Hemotek feeding unit. At the end of the feeding period, fleas were screened for signs of attempted feeding and persistence of blockage, and blood meal reservoirs were screened for bacterial transmission by CFU count (as above). Fleas were categorized as still blocked (and transmitted), still blocked (did not transmit), fed (cleared blockage), or did not attempt to feed. Following feeding, fleas were returned to the housing capsule, placed in the 21°C/75% RH incubator, and were either dissected or monitored for survival without any additional opportunities to feed.

### Estimated transmission efficiency

Transmission efficiency (maximum likelihood estimation or MLE) per individual flea was calculated based on the results of three independent mass transmission experiments using Microsoft Excel Add-In PooledInfRate version 4.0 software that has been used previously to determine cat flea transmission efficiency [33]. The estimation is based on the number of blood meal reservoirs exposed to a potentially infectious flea population, the number of infected fleas that fed on the feeding unit, and whether or not bacteria were recovered from the feeding unit at the end of the feeding period (successful transmission). This calculation requires that 1) at least one infected flea fed on a given day; and 2) the three feeding events were not all positive for bacterial transmission. In cases where both of these conditions were not met, the estimated transmission efficiency was unable to be calculated. The number of fleas that fed in mass transmission experiments that were still infected was calculated based on the weekly infection rate determinations described above.

### Flea dissection and microscopy

Fleas were placed in 20 μl of PBS on a glass microscope slide and dissected with a set of fine forceps. The flea exoskeleton was removed and in most cases an 18 x 18 mm glass cover slip was gently placed over the top of the digestive tract. Images of flea digestive tracts and bacterial biofilms were obtained using a Nikon Eclipse E800 microscope. Fluorescent images of bacterial biofilms and flea digestive tract F-actin were observed with the B-2A (450–490 EX) and the G-2E/C (540/25 EX) fluorescent filters (Nikon). Measurements of the width of the base of the esophagus, the proventriculus, and the muscle layer surrounding the proventriculus were performed using Olympus cellSens imaging software on randomized, coded (blinded) images. All graphically represented measurements were from digestive tracts where a cover slip was applied. Whole fleas were observed and imaged with a Nikon SMZ 1500 dissection microscope. All pictures were obtained with an Olympus DP72 camera and cellSens software.

### Actin staining

Digestive tracts dissected from female *C*. *felis* and *X*. *cheopis* were transferred to a 1.5 ml tube, fixed with 4% formaldehyde for 1 hour, permeabilized with 0.5% Triton X-100 for 15 minutes, blocked with 3% bovine serum albumin for 1 hour, and stained with Alexa Fluor 594 Phalloidin (Life Technologies) for 20 minutes per the manufacturer’s instructions. Following each step, digestive tracts were washed 3 times in PBS. Stained digestive tracts were mounted with ProLong Gold anti-fade reagent (Life Technologies).

### Statistical analysis

All analyses (except estimated transmission efficiency) were performed using SigmaPlot v12.5 (Systat Software Inc., San Jose, Ca.) or GraphPad Prism 6 (GraphPad Software Inc., La Jolla, Ca.). Three independent infection experiments were done for each of the feeding-frequency groups. Statistical tests used and relevant *p* values are indicated in the figure legends.

## Results

### Feeding frequency influences colonization of the cat flea digestive tract by *Y*. *pestis*

**Fig 1:**
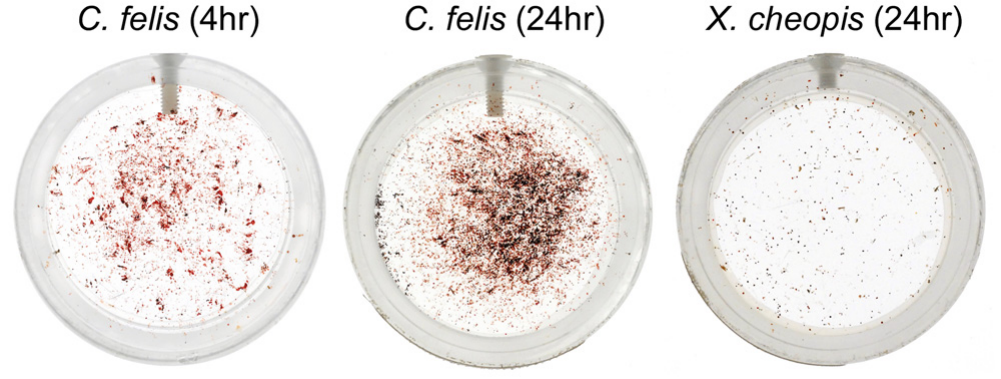
Cat fleas feed more often than rat fleas when given unrestricted access to blood. 50 male and 50 female *C*. *felis* or *X*. *cheopis* fleas were starved for 5 days, placed into a clean capsule, and allowed to feed for either 4 or 24 hours on defibrinated sheep blood in an artificial membrane system. Images show the amount of fecal material that soiled the bottom of the capsule at the end of the feeding period.

The feeding frequency of *C*. *felis* is markedly different from certain fleas that are well-established vectors of *Y*. *pestis* (*e*. *g*. *X*. *cheopis* and *O*. *montana)*. As a demonstration of this, 100 *C*. *felis* and *X*. *cheopis* fleas were starved for 5 days, and then allowed to feed for either 4 or 24 hours on sheep blood. Greater than 90% of fleas of both species took at least one blood meal during the experiments. In the artificial membrane feeding system used, fleas feed from below, and fecal material drops down to soil the bottom of the housing capsule. Images of the capsules at the end of the feeding period provide a comparative estimation of the amount of blood consumed and defecated. Cat fleas produced copious amounts of fecal material relative to *X*. *cheopis* over both a 4- and 24-hour feeding period (Fig 1). Based on the amount of fecal material produced by *C*. *felis*, cat fleas ingest and passage blood through their digestive tract much more frequently than *X*. *cheopis*. These results are consistent with previous observations of cat flea feeding behavior [44] and are shown to contrast the differences in feeding behavior between the two flea species.

**Fig 2:**
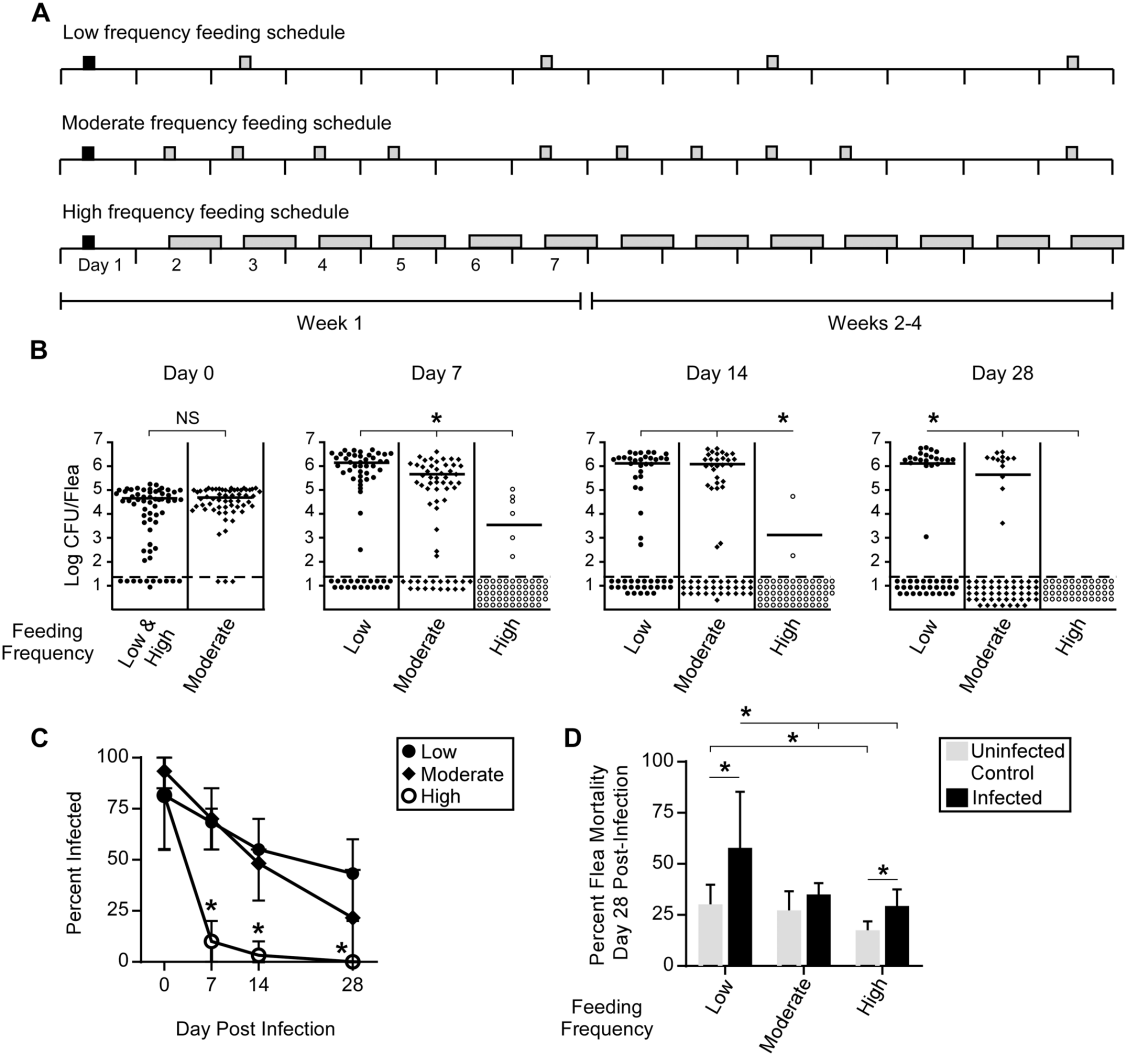
Feeding frequency influences colonization of the cat flea digestive tract by *Y*. *pestis*. Fleas that took a single infectious blood meal that contained ~ 2x10^8^ CFU/ml of *Y*. *pestis* were subsequently provided uninfected maintenance feeds at low frequency (twice weekly for 4h), moderate frequency (five times weekly for 4h), or high frequency (daily for 18-22h) according to the schedule shown in (**A**). Black and grey bars indicate the infectious and maintenance blood meals, respectively; and the width of the bar corresponds to the 4h or 18-22h feeding period. (**B**) The bacterial load in individual fleas from the low-, moderate-, and high-frequency groups was determined in samples of 10–20 fleas collected immediately after the 4h infectious blood meal (day 0) or at 7–28 days after infection. The low- and high-frequency groups fed on the same infectious blood meal prior to segregating them. Horizontal bars represent the mean CFU/flea; dotted lines indicate the limit of detection (40 CFU). The cumulative results of three independent experiments are shown (Day 0, n = 60; Day 7, n = 60; Day 14, n = 54, 60, 60); Day 28 n = 50, 60, 40). *p <0.05 by Student’s t-test (Day 0) or by Kruskal-Wallis test with Dunn’s multiple comparison (Day 7–28). (**C**) Infection rate (percentage of fleas from which CFUs were recovered) at different times after infection. The mean and range of 3 experiments are indicated; *p <0.0166 by chi-square test with Bonferonni’s post-test. (**D**) Cumulative mortality of infected fleas and uninfected control fleas at the end of the 28-day experiments. The mean and standard deviation of 2–3 experiments with each of the three feeding frequency groups are indicated (low, n = 312, 310; moderate, n = 292, 276; high, n = 184, 214; cumulative number of infected fleas and uninfected control fleas, respectively). *p <0.0055 by chi-square test with Bonferonni post-test; NS = not significant.

To determine how differences in feeding frequency might influence colonization of the cat flea digestive tract, groups of cat fleas were infected by allowing them to feed on blood containing ~2x10^8^ CFU/ml of *Y*. *pestis* KIM6+ (pAcGFP1). Fleas that took an infectious blood meal were placed into one of three different groups that were provided uninfected maintenance blood meals twice a week for 4 hours (low frequency), five times a week for 4 hours (moderate frequency), or daily for 18–22 hours (high frequency) over a period of one month (Fig 2A). As expected, the initial infectious dose was equivalent for all three groups (Fig 2B, day 0). However, one week after infection, cat fleas fed at low or moderate frequency had significantly greater infection prevalence (~70%) and bacterial loads than fleas fed at high frequency (Fig 2C and 2D). The majority of cat fleas allowed to feed daily (high-frequency group) had cleared the digestive tract of infection, with only 10% of fleas remaining infected after one week, and these individuals had significantly lower bacterial burdens than infected fleas in the other two groups (Fig 2B, day 7). By day 14, only 2 of the 60 fleas allowed daily maintenance feeds remained infected. Cat fleas fed at low or moderate frequency had similar *Y*. *pestis* infection rates and bacterial loads throughout most of the infection. By day 28, on average, 22–43% of *C*. *felis* that fed at low or moderate frequency remained infected with *Y*. *pestis*, whereas all cat fleas that fed at high frequency cleared the infection (Fig 2C). Therefore, cat flea feeding frequency significantly influences *Y*. *pestis* colonization of the digestive tract. The uninfected control flea groups also revealed a fitness cost associated with decreasing the cat flea’s preferred frequent feeding behavior. Control fleas fed daily had a significantly lower mortality rate (~16%) over the 28-day experiments than fleas fed only twice per week (~29%; Fig 2D). Maintenance blood meals of the group fed at high frequency were supplemented with carbenicillin in this study, but this had no discernable effect on flea fitness (Fig 2D).

### Cat flea feeding frequency influences the development and severity of proventricular infection

**Fig 3:**
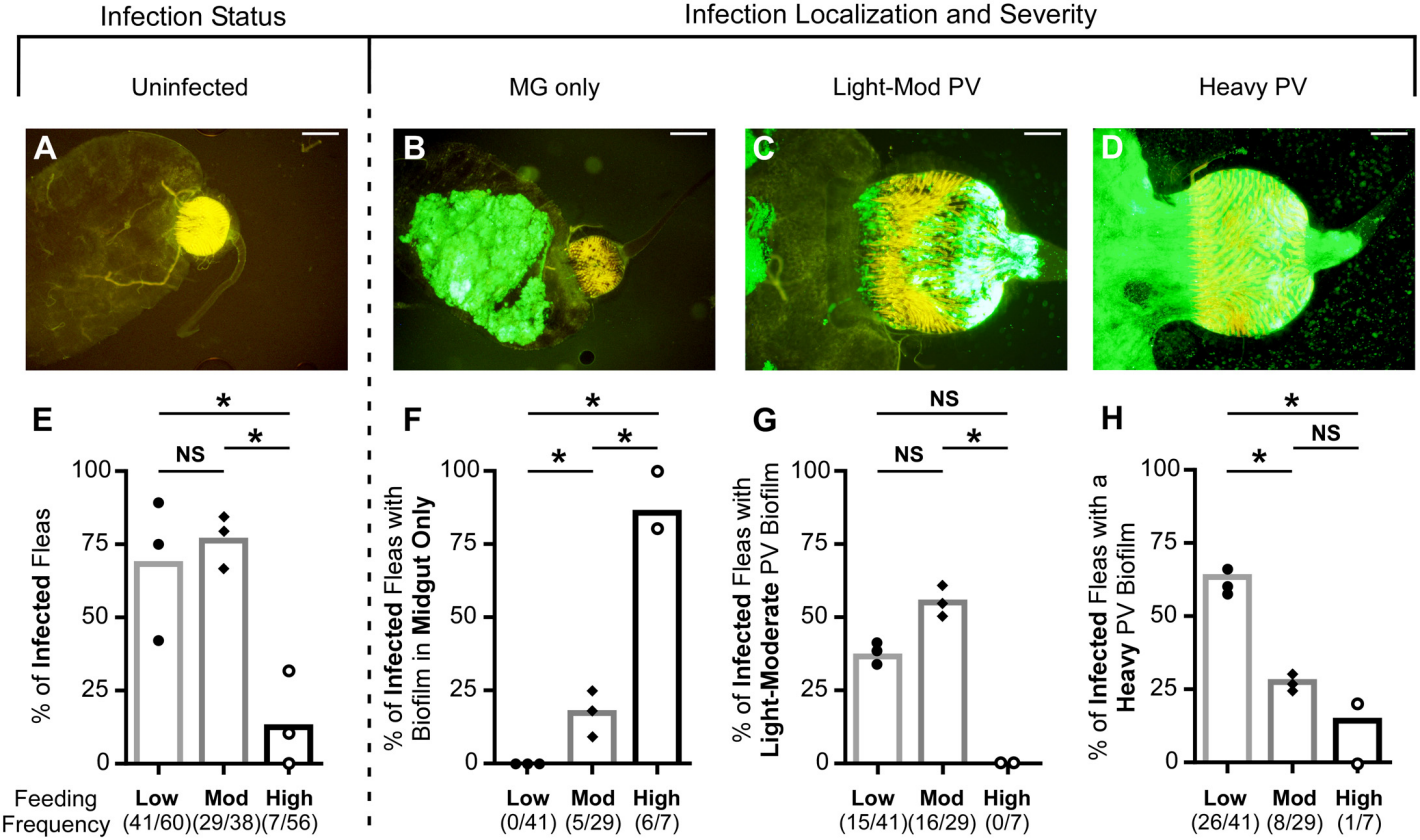
Cat flea feeding frequency influences severity of proventricular infection. 38–60 mixed-sex cat fleas were dissected 7 days after a blood meal containing ~2x10^8^ *Y*. *pestis* KIM6+ (pAcGFP1) CFU/ml and the digestive tracts were visualized with fluorescence microscopy for the presence and localization of a *Y*. *pestis* biofilm. Representative images of an uninfected flea (**A**; no visible bacteria in the digestive tract; the spines of the proventriculus [PV] autofluoresce yellow); a flea with an infection only in the midgut (MG) (**B**; no green-fluorescent bacteria observable in the PV); and fleas with a light-moderate PV infection (**C**; <75% bacterial coverage of the PV) or a heavy PV infection (**D**; ≥75% bacterial coverage of the PV). Areas that appear white are due to fluorescence overexposure when acquiring the image. Scale bars = 100 μm (A, B) or 50 μm (C, D). (**E-H**) The percentage of infected fleas (E), and of those, the percentages with midgut-only infections (F), and with midgut plus light-moderate (G) or heavy (H) PV infections in three independent infection experiments for the three feeding-frequency groups are shown; bars indicate the mean. Each symbol represents an individual experiment. One experiment with the high-frequency group had no infected fleas at 7 days (thus only two symbols shown for this group in F-H). Numbers in parentheses indicate the number of positives/total number of fleas examined. * *p* < 0.0166 by chi-square test with Bonferonni post-test; NS = not significant.

A major transmission mechanism of *Y*. *pestis* depends on the formation of sufficient bacterial biofilm in the proventriculus to impede normal blood flow during feeding. To determine the capacity of *C*. *felis* to support a proventricular infection, a total of 38–60 cat fleas maintained at the low-, moderate-, or high-frequency feeding schedules were dissected one week after infection with *Y*. *pestis* KIM6+ (pAcGFP1). Flea digestive tracts were visualized by fluorescence microscopy and categorized as uninfected (no fluorescent biofilm detected, Fig 3A) or infected, with biofilm localized to the midgut only (Fig 3B), or to both the midgut and proventriculus. Proventricular infections were scored as either light-moderate (the biofilm covered < 75% of the proventricular surface area; Fig 3C) or heavy (the biofilm covered ≥75% of the proventriculus; Fig 3D). Consistent with the bacterial CFU plating data (Fig 2B), only 12% of fleas fed at high frequency were still infected (green-fluorescent bacteria observed), compared to ~70% of fleas fed at low or moderate frequency (Fig 3E). By this 7-day time point, infection was characterized by large aggregates of GFP-positive bacteria (Fig 3B–3D). The percentage of infected fleas in which the bacterial aggregates had localized only to the midgut was significantly higher in the group fed at high frequency (~85%) than in the group that fed at moderate frequency (17%); and none of the fleas in the low frequency feeding group had a midgut-only infection–the proventriculus was always colonized to some extent (Fig 3F). The relative amount of proventricular biofilm also differed among the three groups. Between 37 and 55% of cat fleas fed at low to moderate frequency had developed a light-moderate proventricular infection; 63% of infected fleas fed at low frequency and 28% of infected fleas fed at moderate frequency had a heavily colonized proventriculus one week after infection (Fig 3G and 3H). In sharp contrast, only 7 of 56 fleas in the high-frequency feeding group were infected, and only one of these infections had localized to the proventriculus. Thus, the severity of proventricular infection is inversely correlated with feeding frequency.

### Cat flea proventricular blockage rate is strongly influenced by feeding frequency after the infectious blood meal

**Fig 4:**
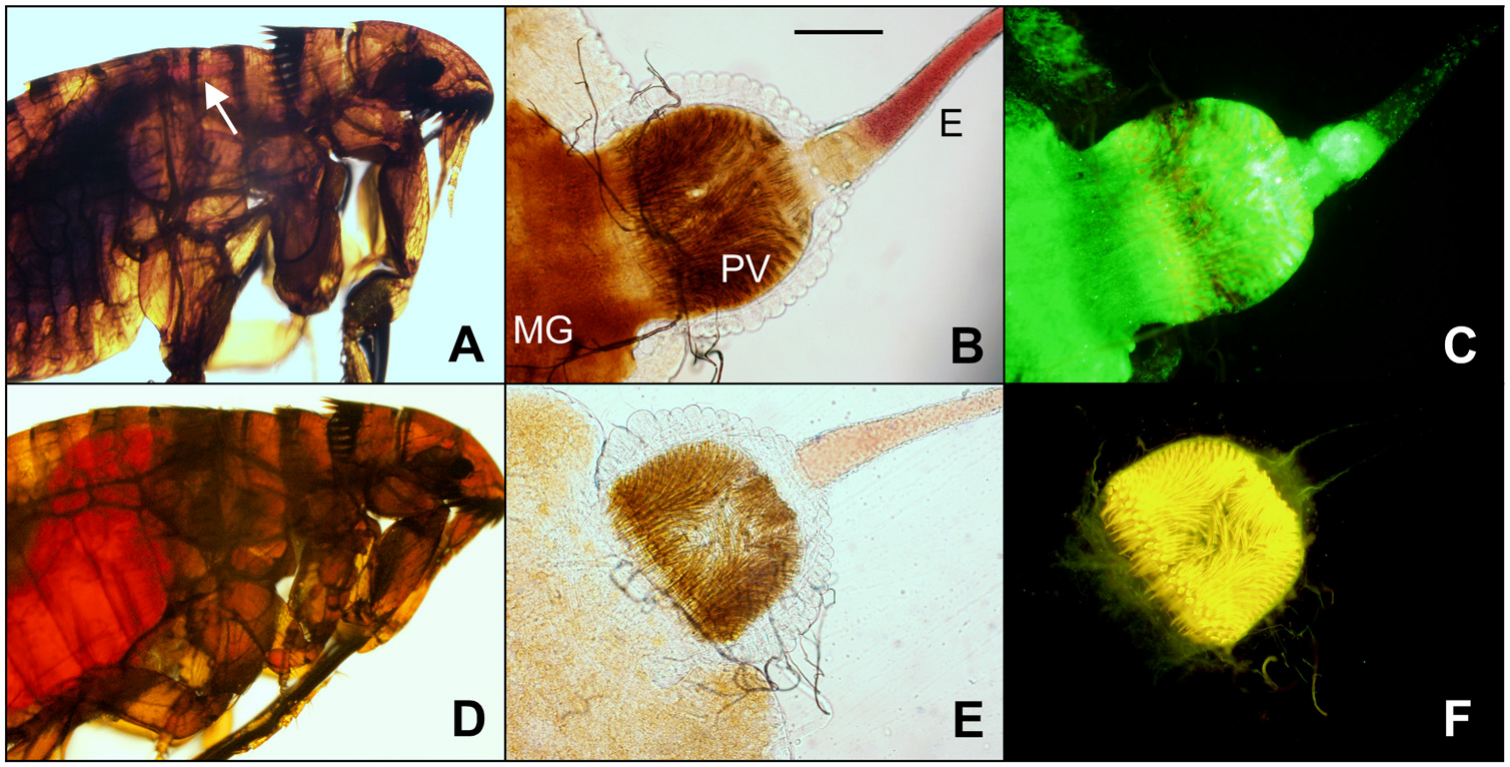
*Y*. *pestis* is capable of producing proventricular blockage in *C*. *felis*. **(A**) Female cat flea blocked with *Y*. *pestis* expressing GFP, examined immediately after a feeding attempt. Fresh red blood is present in the esophagus only (arrow), with none in the midgut, which contains dark, digested blood from previous blood meals. (**B**) Bright field and (**C**) fluorescent microscopy images of the dissected digestive tract of the same blocked flea, showing the dense GFP+ bacterial mass that fills and blocks the proventriculus (PV) and also extends into the esophagus (E) and midgut (MG). Fresh red blood that was blocked from entering the midgut is still observable in the esophagus. (**D-F**) The same image series for an uninfected cat flea, which has fresh red blood in the midgut and an absence of a dark bacterial mass in the proventriculus. Scale bar = 100 μm.

Given the heavy proventricular infection observed in some fleas, it seemed plausible that they could eventually become blocked. Fleas were monitored for the development of proventricular blockage over the 28-day infection period. Immediately following each maintenance feed, fleas were individually examined microscopically for blockage. Cat fleas identified as blocked consistently displayed red blood in the esophagus as well as dark midgut contents, likely consisting of a combination of partially digested blood from previous blood meals and bacterial aggregates (Fig 4A). Of the 58 fleas diagnosed as blocked, 44 were dissected to confirm the presence of a bacterial biofilm that filled the proventriculus. Even after dissection, red blood could be seen in the esophagus and the proventriculus was obstructed by a bacterial mass that occasionally extended outward, beyond the base of the esophagus (Fig 4B and 4C).

During the four-week period following an infectious blood meal, up to 15.2% of cat fleas developed complete blockage of the proventriculus (Table 1). However, the blockage rate correlated strongly with decreased frequency of maintenance feeds. None of the fleas permitted to feed daily after infection became blocked, whereas ~12% of fleas in the low feeding-frequency group became blocked. Fleas in the moderate-frequency group had an intermediate blockage rate of ~7%. Fleas became blocked as early as day 6 post-infection and as late as day 28 in both the low and moderate frequency groups, with a mean time to blockage of 16.5 and 14 days, respectively, which was not significantly different (Table 1). The mean time to blockage for all blocked fleas was 15.8 ± 6.8 days (n = 58, 34♀/24♂,). Our data demonstrate that cat fleas feeding at their normal high-frequency (daily) rate for extended periods of time (18–22 hours) are unlikely to become infected and blocked by *Y*. *pestis*, but they can become blocked if access to daily blood meals from an uninfected host is restricted.

**Table 1:** Summary of *C*. *felis* infection experiments.

Flea group	*Y*. *pestis* CFU/ml in infectious blood meal*	Fleas (n)	% Blocked fleas (n)	Mean time to block (days)
**Low-frequency maintenance feeding schedule:**				
Expt 1	2.3x10^8^- 4.9x10^8^	105	6.7% (7)	16.9
Expt 2	1.3x10^8^- 4.5x10^8^	102	14.7% (15)	14.4
Expt 3	2.5x10^8^- 4.4x10^8^	105	15.2% (16)	18.4
**mean**	**2x10^8^- 4.6x10^8^**	**104**	**12.2%**^†^^§^	**16.5**
**Moderate-frequency maintenance feeding schedule:**				
Expt 1	1.3x10^8^- 8.4x10^8^	96	4.2% (4)	12
Expt 2	2.3x10^8^- 6.1x10^8^	108	9.3% (10)	16
Expt 3	2.3x10^8^- 5.7x10^8^	88	6.8% (6)	12.2
**mean**	**2x10^8^- 6.7x10^8^**	**97**	**6.8%**^†^	**14**
**High-frequency maintenance feeding schedule:**				
Expt 1**	2.3x10^8^- 4.9x10^8^	104	0% (0)	NA
Expt 2	1.3x10^8^- 4.5x10^8^	92	0% (0)	NA
Expt 3	2.5x10^8^- 4.4x10^8^	92	0% (0)	NA
**mean**	**2x10^8^- 4.6x10^8^**	**96**	**0%**	**NA**

*Range indicates the CFU/ml determined immediately after adding the bacteria to the blood meal and again after the 4-hour feeding period.

**Expt 1 with the high-frequency group was terminated after 22 days.

^†^*p* < 0.0001 compared to the high-feeding frequency group;

^§^*p* = 0.026 compared to moderate-frequency group by chi-square test

Mean time to blockage was not significantly different between groups by chi-square test

Proventricular biofilm interferes with normal blood feeding and fleas that are completely blocked rapidly starve. Thus, increased mortality is an indirect indicator of the severity of proventricular infection. The mortality rate of infected fleas correlated with their observed rates of heavy proventricular infection and complete blockage, with fleas in the low-frequency feeding group having the highest rates of all three (Figs 2D and 3, Table 1). For example, ~60% of fleas randomly selected from the low-frequency group had a heavy proventricular biofilm infection by day 7 (Fig 3D–3H), which may impede blood feeding enough to effect fitness. Overall, the data indicate that the severity of proventricular infection with *Y*. *pestis* is inversely correlated with feeding frequency.

### Transmission potential of *Y*. *pestis*-infected cat fleas

**Fig 5:**
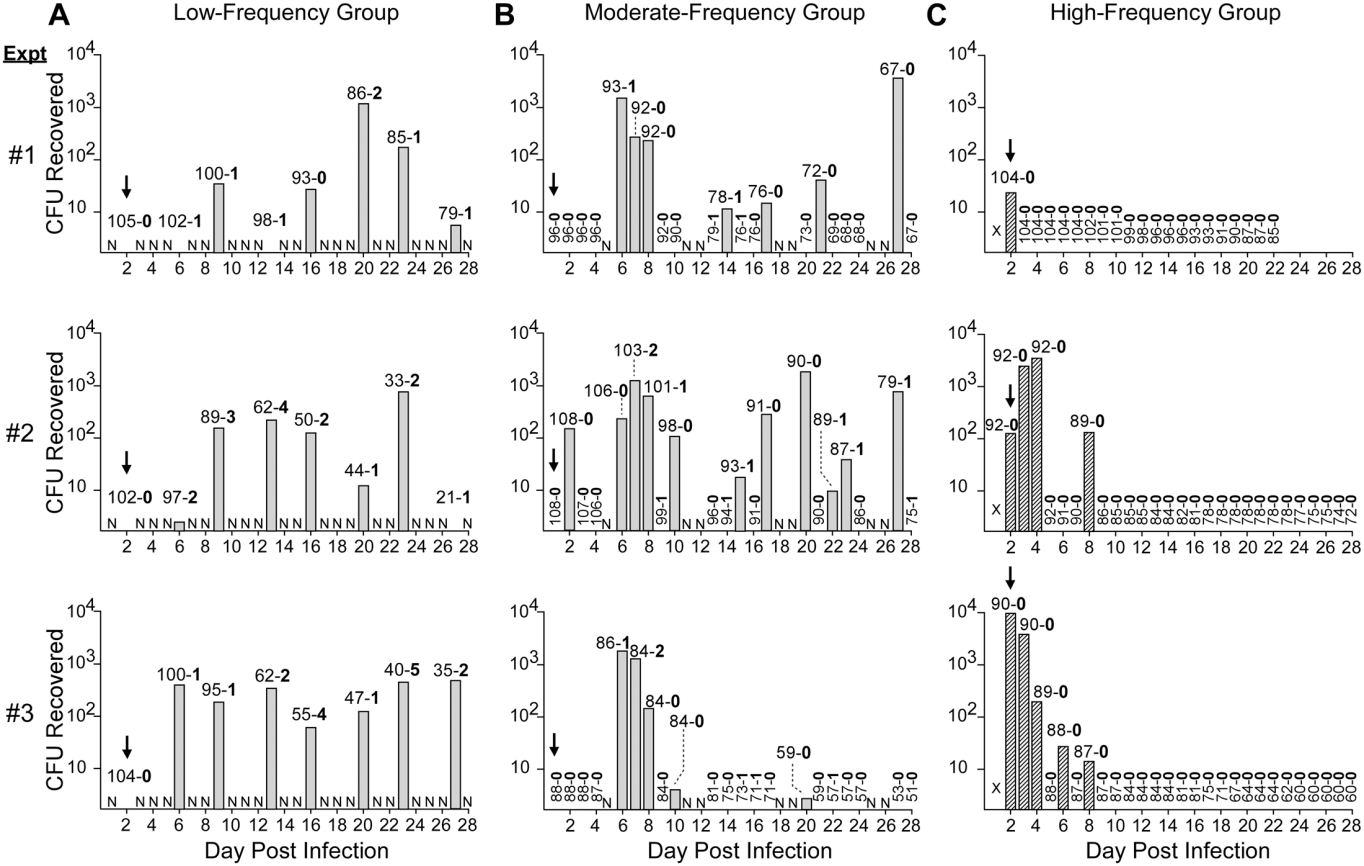
Cat fleas transmit *Y*. *pestis* after the early phase at an efficiency that correlates with feeding frequency and proventricular blockage rate. The number of *Y*. *pestis* CFUs recovered from the blood meal reservoir after maintenance feeds by ~100 cat fleas fed for 28 days at either **A)** low frequency (twice a week for 4 hours), **B)** moderate frequency (five times a week for 4 hours), or **C)** high frequency (daily for 18–22 hours) from 3 independent experiments (top to bottom, experiments 1 through 3). Blood was plated after a 4-hour feeding period for the low- and moderate-frequency groups (grey bars) or after an 18–22 hour feeding period for the high-frequency group (hatched bars). All infected flea groups initiated their maintenance feeding schedules 24 hours after the infectious blood meal. The numbers above each bar indicate the number of non-blocked and blocked fleas that fed on that day (# non-blocked (dash) **# blocked**). Blocked fleas were removed from the feeding pool upon identification. N, not fed on the indicated day; arrows indicate expected peak occurrence of early-phase transmission (EPT); X indicates that feeding began on day 1 post-infection but extended into day 2 (see Fig 2A). The results of three independent experiments with each group are shown.

Due to the capacity for cat fleas to develop heavy proventricular infections that can lead to blockage, we wanted to assess the cat flea’s competence as a vector. To do this, bacterial transmission was screened in a “mass” transmission experimental model. After each regularly scheduled maintenance feed on sterile blood by groups of ~100 infected fleas, the entirety of the feeding unit’s blood reservoir was plated for recovery of viable *Y*. *pestis*. Cat fleas fed at low to moderate frequency were capable of transmitting throughout the 28-day period (Fig 5A and 5B). However, fleas allowed to feed at high frequency only transmitted over the first 8 days of infection, with the majority of transmission events occurring during the first 4 days (Fig 5C). The percentage of feeding days in which transmission occurred was inversely correlated with feeding frequency (high to low feeding frequency: 12.8%, 36.5%, and 75% of test days with positive transmission results).

For cat fleas fed at low to moderate frequency, early-phase transmission (EPT, occurring on the first maintenance feed following infection; *i*.*e*. day 2 for the low-frequency group and day 1 for the moderate-frequency group) was not detected (Fig 5, arrows). In 1 of 6 experiments with these two groups of fleas, transmission occurred on the second feeding after the infectious blood meal, but otherwise transmission began on day 6, concomitant with the first detection of blockage (Fig 5B, experiment 2). These results are consistent with previous reports that cat fleas are relatively inefficient transmitters during the early phase [26,33]. In contrast to the early phase, *Y*. *pestis* was transmitted in ~89% and ~36% of feeding days during weeks 2–4 of infection by low-frequency and moderate-frequency group fleas, respectively, during the period in which blocked fleas were diagnosed.

Transmission by cat fleas fed at high frequency exhibited a distinctly different pattern. Fleas in this group transmitted predominantly during the first three days after the infectious blood meal, with no transmission after day 8 (Fig 5C). The time frame suggests this represents EPT; however this does not conform to the traditional definition of EPT occurring predominately during the first feeding after the infectious blood meal, with a decrease in transmission upon subsequent blood meals. Furthermore, EPT by fleas in the high-frequency group appeared to require the long 18–22 hour feeding period, because EPT was not detected in the other groups that were infected identically, but allowed to feed for only 4 hours (Fig 2A).

From 2 to 3780 CFU were recovered from the mass transmission blood meals for the low- and moderate-frequency group cat fleas. However, these numbers include CFU actually transmitted plus any post-transmission bacterial multiplication in the feeding reservoir during the 4-hour feeding period. We determined that *Y*. *pestis* numbers increase 2.5- to 7-fold during a 4-hour incubation in defibrinated sheep blood at 37°C, depending on the growth phase of the bacteria used to inoculate the sterile blood (Table 1). Thus, it is likely that our values for the number of CFUs transmitted (Fig 5) are artificially high. This caveat is even more pertinent to the results from fleas in the high-frequency fed group, which were allowed to feed for 18–22 hours before blood was collected for CFU plating. A range of 17 to >10,000 CFUs were recovered from transmission experiments with the high-frequency group, but the majority presumably reflect extensive bacterial growth in the blood meal during the overnight feeding period. Thus, the significant difference in the length of the feeding period make it impossible to quantitatively compare the actual numbers of CFU transmitted by the high-frequency vs. the low- and moderate-frequency groups (indicated by hatched bars in Fig 5C).

To further assess the competence of *C*. *felis* as a vector, transmission efficiency (the probability that an individual infected flea will transmit *Y*. *pestis* during a bite) was evaluated by MLE statistical analysis. Results from time points (days after infection) common to all 3 feeding schedules and encompassing both early-phase and later biofilm-mediated transmission were analyzed. Overall, transmission efficiencies for the cat flea were low, ranging from 0–3.76% across all three feeding-frequency groups (Table 2). For fleas in the low- and moderate-frequency feeding groups, transmission efficiency increased with time post-infection. In contrast, fleas that fed at high frequency always transmitted on day 2 post-infection, once in 3 experiments on day 6, but never on days 13 or 20 (Table 2). Lack of transmission by this group of fleas at later time points reflects the very low infection rates after one week post-infection (Fig 2, Table 2). An estimated transmission efficiency (3.76%) could only be calculated for day 6 post-infection. Estimated transmission efficiencies could not be calculated for day 2 because all pools transmitted, or for days 13 and 20 because no transmission was detected and most fleas had already cleared the infection.

**Table 2:** Transmission efficiency of infected cat fleas.

Transmission results at different times after the infectious blood meal*				
Flea group	Day 2	Day 6	Day 13	Day 20
**Low-frequency maintenance feeding schedule:**				
Expt 1	– (57)	– (56)	– (29)	+ (26)
Expt 2	– (91)	+ (74)	+ (46)	+ (30)
Expt 3	– (105)	+ (75)	+ (41)	+ (27)
Estimated Transmission Efficiency (MLE)** (95% CI)†	**0 (0–.96)**	**1.18 (.25–6.44)**	**2.12 (.44–11.76)**	** ND**
**Moderate-frequency maintenance/ feeding schedule:**				
Expt 1	– (96)	+ (65)	– (36)	– (37)
Expt 2	+ (102)	+ (90)	– (52)	+ (63)
Expt 3	– (74)	+ (47)	– (36)	+ (41)
Estimated Transmission Efficiency (MLE)** (95% CI)	**.36 (.02–2.17)**	** ND**	**0 (0–1.96)**	**1.72 (.36–9.39)**
**High-frequency maintenance feeding schedule:**				
Expt 1	+ (57)	– (0)	– (0)	– (0)
Expt 2	+ (82)	– (9)	– (8)	– (3)
Expt 3	+ (90)	+ (17)	– (0)	– (0)
Estimated Transmission Efficiency (MLE)** (95% CI)	** ND**	**3.76 (.26–29.26)**	** ND**	** ND**

*+/- indicates whether or not bacteria were transmitted to the blood meal reservoir; number in parentheses is the estimated number of infected fleas that fed, based on data shown in Fig 2 (see Methods for details)

** MLE; maximum likelihood estimation; ND, not able to calculate MLE because all experiments were positive for transmission or because all fleas were uninfected in more than one of the three experiments

^†^ 95% confidence interval

It is important to note that all blocked fleas documented on the mass transmission graphs represent a distinct, individual flea becoming blocked (Fig 5A–5C). Fleas that became blocked only participated in transmission up until the day they were diagnosed, at which point they were removed from the population in order to be analyzed separately. This likely altered the subsequent transmission potential of the populations, as the fleas most likely to transmit were removed from mass transmission pools, potentially resulting in conservative estimates of biofilm-mediated transmission efficiency.

In summary, the mass transmission data indicate that cat fleas are competent vectors for *Y*. *pestis* and their transmission efficiency increases over time when fed at low to moderate frequency. The converse is true for cat fleas fed at high frequency, as their transmission efficiency declined rapidly after the first week of infection.

### Individual blocked cat fleas transmit *Y*. *pestis*

**Fig 6:**
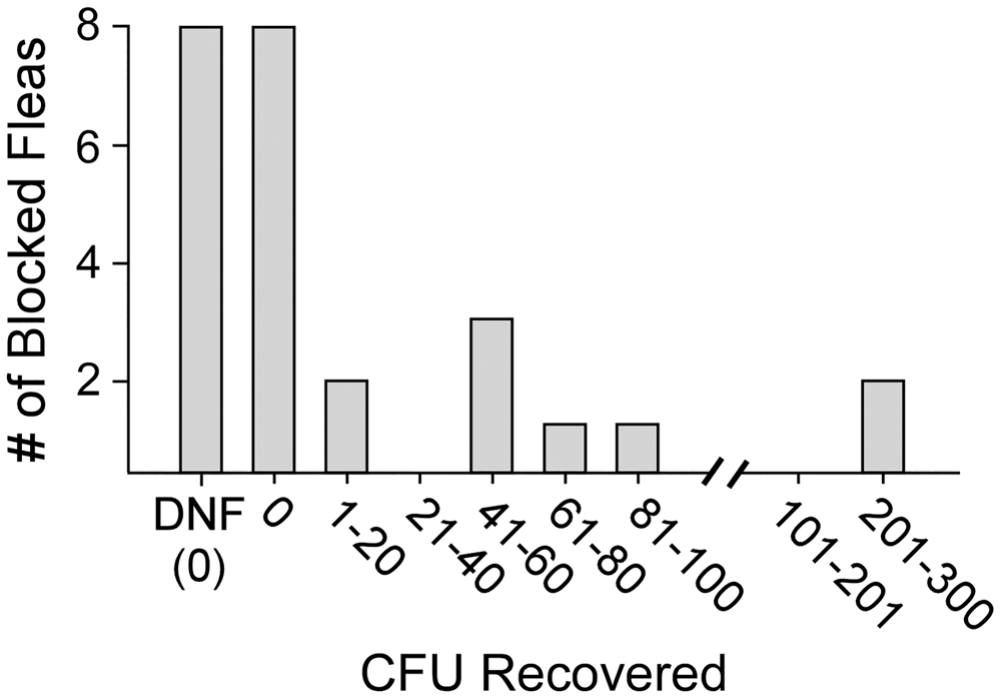
Transmission by individual blocked cat fleas. Frequency distribution of CFUs recovered from blood meal reservoirs fed on by individual blocked cat fleas (n = 25) after a 4-hour feeding period. DNF = did not attempt to feed.

A portion of the blocked fleas that were diagnosed during the mass transmission trials (Table 1, Fig 4) were removed and monitored separately for survival and transmission capability. Individual blocked fleas were given access to a separate blood meal reservoir for 4 hours the day after their diagnosis. Of 25 blocked fleas examined, 17 showed evidence of having attempted to feed (fresh red blood in the esophagus and/or midgut), and of these, 9 fleas transmitted *Y*. *pestis* (19–262 CFUs recovered; median = 49), with 7 out of the 9 transmission events yielding <100 CFU (Fig 6).

As described above, the actual numbers of bacteria transmitted may be lower than the number of CFUs recovered, as bacteria transmitted early during the 4-hour feeding period likely replicated, resulting in an estimated increase of 2.5 to 7-fold in the blood meal reservoir. Eight blocked fleas did not show evidence of feeding and did not transmit bacteria. Of the 17 fleas that showed evidence of feeding, 4 appeared to have either completely or partially cleared the biofilm blockage, because fresh red blood was present in the midgut. Those blocked fleas that cleared the blockage and acquired a new blood meal did not transmit bacteria. Blocked cat fleas died within 1–5 days after being diagnosed as blocked, on average, surviving 2.93 ± 1.14 days (n = 14, 10♀/4♂). Fleas that cleared their blockage were not included in survival analysis.

### Differences in foregut anatomy between *C*. *felis* and *X*. *cheopis* that may affect vector competence

**Fig 7:**
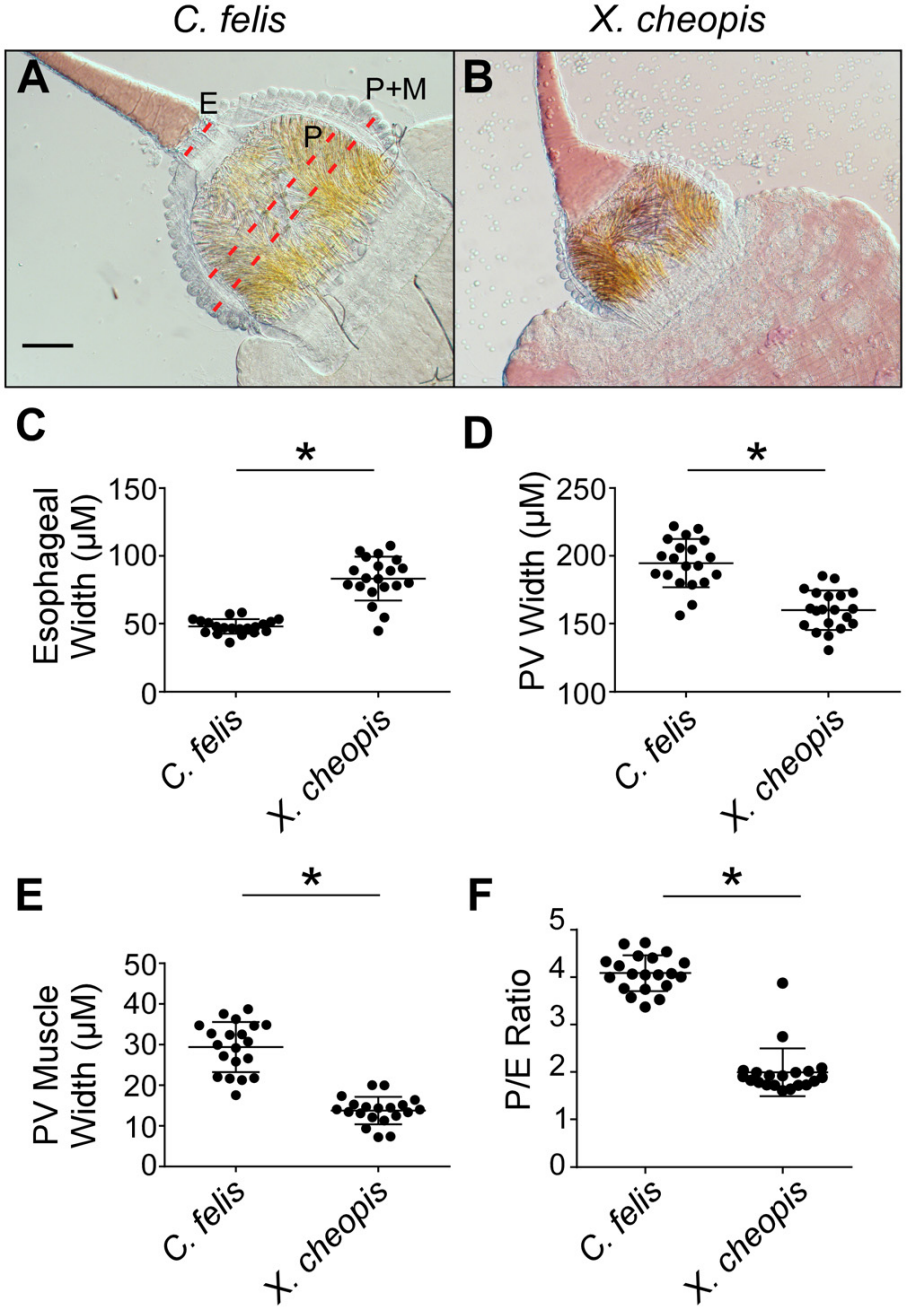
The cat flea has a narrower esophagus and thicker proventricular musculature than the rat flea, *X*. *cheopis*. Digestive tracts dissected from recently fed, uninfected female *C*. *felis* (**A**) or *X*. *cheopis* (**B**) fleas in 20 μl of PBS were overlaid with a cover slip and the base of the esophagus (E) and the widest part of the proventriculus (P), and the proventriculus plus surrounding musculature (P+M) were measured. Dotted red lines indicate the location where measurements were taken. (**C-E**) Width of the esophagus, proventriculus, and proventricular muscle layer (P+M–P)/2) of individual fleas. (**F**) Ratio of proventricular width to esophageal width (P/E). Horizontal bars indicate the mean and error bars the standard deviation (n = 20). *p > 0.0001 by Student’s t-test. Scale bar = 50 μm.

Hydrodynamic forces generated during blood feeding likely affect colonization of the proventriculus and regurgitative transmission of *Y*. *pestis* [45]. Because these forces depend on physical anatomy of the foregut, which may vary among flea species, we compared esophageal and proventricular dimensions of uninfected female *C*. *felis* and *X*. *cheopis*. The proventriculus and the muscle layer surrounding it were both significantly wider in *C*. *felis* than in *X*. *cheopis*, whereas the cat flea esophagus is narrower (~48 μm) than that of *X*. *cheopis* (~83 μm) (Fig 7). The proventriculus::esophagus (P/E) width ratio for cat fleas was ~4, twice that of *X*. *cheopis* (Fig 7F).

**Fig 8:**
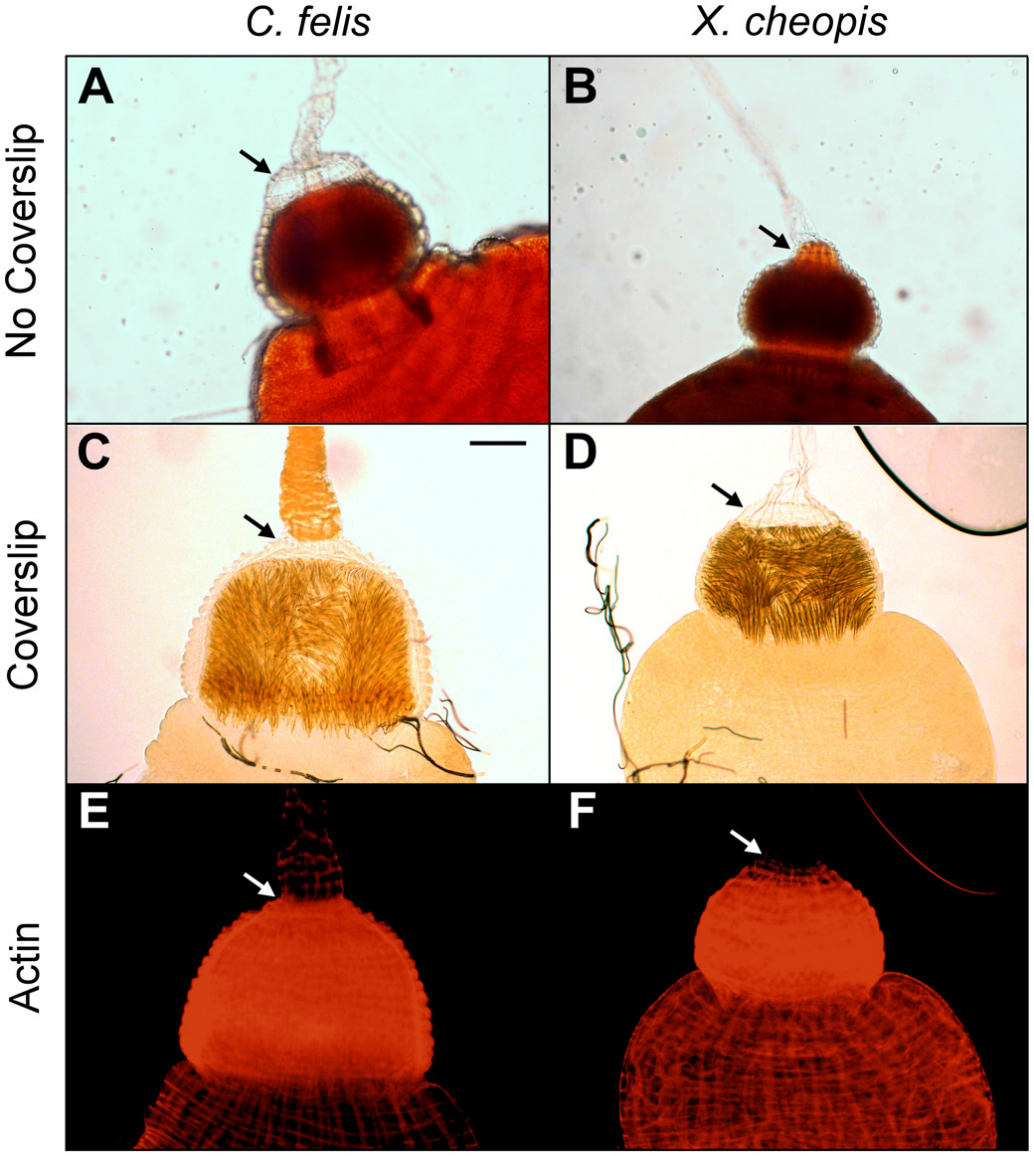
Proventricular musculature surrounds and constricts the base of the esophagus of *C*. *felis*, but not of *X*. *cheopis*. Digestive tracts dissected from uninfected female *C*. *felis* **(A)** or *X*. *cheopis* **(B)** fleas in 20 μl of PBS were photographed without the application of a cover slip. Arrows indicate a layer of muscle surrounding the base of the esophagus that is present in *C*. *felis* but absent in *X*. *cheopis*. **(C-F)** Actin stains of fixed, permeablized *C*. *felis* **(C, E)** and *X*. *cheopis* **(D, F)** digestive tracts were visualized by bright field and fluorescence microscopy (red fluorescence = actin). Representative images of 14–18 stained digestive tracts from 2 independent experiments are shown. Scale bar = 50 μm.

Uninfected midguts were also imaged without application of a coverslip, because the coverslip weight potentially affected width measurements by compressing the lightly-muscled *X*. *cheopis* foregut more than that of *C*. *felis*. Images taken without the application of a coverslip showed similar trends in foregut dimensions between species (Fig 8A and 8B). Interestingly, in these preparations, a thick muscle layer surrounding the base of the esophagus that was absent in the rat flea became obvious (arrow, Fig 8A–8D). The musculature narrows the cat flea esophagus at its base, which was not observed in *X*. *cheopis*. Phalloidin stains of fixed, permeabilized foreguts also showed noticeably less actin, a major component of muscle tissue, surrounding the base of the rat flea esophagus compared to the cat flea (arrow, Fig 8E and 8F).

**Fig 9:**
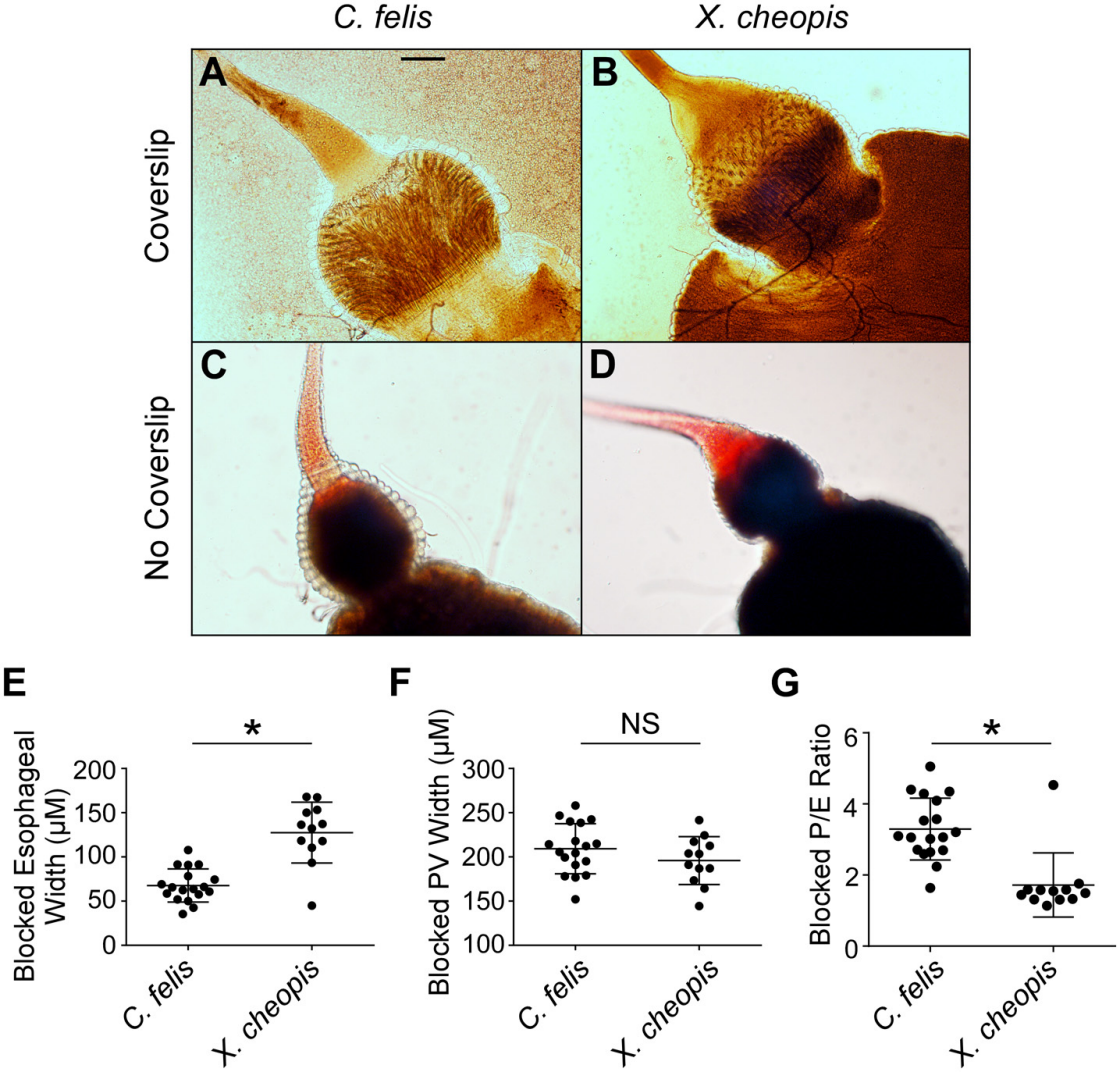
The cat flea esophagus is less prone to blockage-induced distension than the rat flea esophagus. Digestive tracts dissected from blocked female *C*. *felis* (**A**) or *X*. *cheopis* (**B**) fleas in 20 μl of PBS with (**A**, **B**) or without (**C**, **D**) the application of a cover slip. Representative images of 12–18 (with coverslip) or 15 (no coverslip) blocked fleas are shown. The width of the esophagus (**E**) and proventriculus (**F**) of individual blocked fleas was measured, and the proventriculus::esophagus width ratio (P/E) was calculated (**G**). Horizontal bars indicate the mean and error bars the standard deviation (n = 12–18). *p > 0.0001 by Student’s t-test. Scale bar = 50 μm.

We examined whether the thicker muscle layer that surrounds the base of the cat flea esophagus would constrict esophageal expansion typically seen in blocked *X*. *cheopis* fleas. Blocked *X*. *cheopis* fleas dissected immediately after feeding, which filled their esophagus with blood, exhibited increased distension of the base of the esophagus when compared to blocked *C*. *felis* (Fig 9A–9D), and the proventricular biofilm appeared to extend into the posterior portion of the esophagus. The esophageal width of blocked cat fleas (~68 μm) was significantly less than that of blocked rat fleas (~127 μm). The average esophageal width of blocked cat fleas increased by ~41% above uninfected cat fleas, whereas the blocked rat flea esophageal width increased by ~53% (Figs 7C and 9E). While the overall width of a blocked proventriculus was similar between flea species (Fig 9F), the increase in width from uninfected to blocked fleas was three-fold greater in rat fleas (~22.5% increase) compared to cat fleas (~7.5% increase). Consistent with measurements of uninfected fleas, the blocked proventricular/esophageal width ratio also differed significantly between the two species (Fig 9G).

## Discussion

In this study, we have evaluated the vector competence (the capability to transmit *Y*. *pestis*) of the cat flea *C*. *felis*. Three fundamental components of vector competence were assessed: 1) the percentage of fleas that become infected after feeding on bacteremic blood; 2) the percentage of infected fleas that develop a dense proventricular biofilm infection important for biological transmission; and 3) the actual transmission efficiency during a 28-day period after infection. Because the feeding behavior of *C*. *felis* differs markedly from that of the established plague vectors *X*. *cheopis* and *O*. *montana*, we also determined the effects that frequency and length of post-infectious blood meals have on vector competence.

Our data demonstrate that when infected *C*. *felis* are restricted to a feeding pattern typical of *X*. *cheopis*, *Y*. *pestis* can survive, multiply, and block the digestive tract. However, cat fleas will feed much more frequently if given the opportunity, and this feeding pattern significantly decreased vector competence in our experiments. The majority of fleas that were provided daily, prolonged access to maintenance blood meals rapidly cleared their infection, the few chronically infected fleas rarely developed a heavy proventricular infection, and complete blockage was never detected. Cat fleas excrete large amounts of partially digested blood while feeding (Fig 1) [46]. This frequent turnover of the digestive tract contents likely results in complete elimination of *Y*. *pestis* in the feces before the bacteria can multiply and form the large biofilm aggregates that typify a chronic infection. Frequent blood meal acquisition and defecation are also characteristic of *Xenopsylla skrjabini*, a flea that will normally feed several times a day on great gerbils [47]. Infected *X*. *skrjabini* provided daily post-infection blood meals also tended to clear their infections at a high rate, although up to 60% showed evidence of complete or partial blockage within 14 days. However, this study used a particularly high-titer infectious blood meal (1x10^10^ CFU/ml) and the daily maintenance feeding duration was only 2 hours.

Another potential contributing factor is that cat fleas often remained associated with the artificial membrane during maintenance feeds. The combination of frequent ingestion of 37°C blood and close contact with the membrane (surface temperature of 31.5°C) during the 18–22 hour daily feeds may raise the flea body temperature, resulting in decreased or less adherent biofilm formation. Synthesis and export of the extracellular polysaccharide matrix encoded by the *hmsHFRS* operon and required for biofilm formation are temperature-dependent, and rat fleas maintained at >25°C have greatly reduced proventricular blockage rates [43,48]. A previous study that examined *Y*. *pestis* infection of cat fleas reported higher rates of infection and proventricular biofilm development than we observed in fleas provided daily feeds during the first two weeks after infection; however, daily feeds lasted only 5 hours in that study [35].

Infection rates, the development of heavy proventricular infections, and the incidence of complete blockage of the proventriculus were all significantly higher in cat fleas fed twice-weekly (low frequency) or five times per week (moderate frequency) for 4 hours and kept at 21°C between feedings. These rates were inversely correlated with feeding frequency. Fleas fed at low frequency had the highest rates of chronic infection, localization to the proventriculus during infection, and complete blockage. We were able to directly demonstrate complete proventricular blockage of cat fleas in this study, but found it much more difficult to diagnose in *C*. *felis* than in *X*. *cheopis* by the standard screening method (microscopic examination immediately after a feeding attempt and observing fresh red blood in the esophagus only, with none in the midgut). The exoskeleton of *C*. *felis* is much more heavily sclerotized and pigmented, and therefore much less transparent, than that of *X*. *cheopis*. In addition, the pair of respiratory tracheae extending posteriorly from the head are thicker and darker in *C*. *felis* than in *X*. *cheopis* and often obscure the esophagus. The pronotal comb, a set of dark chitinous spines located on the dorsal part of the prothorax, can also block external visualization of portions of the esophagus, particularly when the thorax and abdomen contract due to blockage-induced dehydration. These obscuring features were especially pronounced in male *C*. *felis*, and visualization of blockage required a very bright light source, and often lateral compression of a flea underneath a coverslip. For these reasons, a diagnosis of blockage was confirmed in the majority of cases by dissection to demonstrate that the proventriculus was completely filled with GFP+ *Y*. *pestis* and that fresh blood was present in the esophagus but not the midgut (Fig 4). It has been suggested that up to 50% of infected cat fleas could potentially become blocked based on detection of proventricular biofilm in dissected fleas and increased mortality rates [35]. However, this estimation assumes that all such infected fleas eventually become blocked, which was not observed in our analysis.

Previous studies of transmission of *Y*. *pestis* by cat fleas assessed early-phase transmission (EPT) only and consistently reported that they were very inefficient vectors compared to other flea species during the first week after infection [27,31,33]. We found the same result in our experiments, in which groups of ~100 fleas were fed on sterile blood in an artificial feeding system. No *Y*. *pestis* were recovered from the first blood meal 1–2 days after infection, when EPT would be expected to be highest, for fleas that fed for 4 hours (low- and moderate-frequency groups). Interestingly, *Y*. *pestis* was recovered from the first blood meal initiated 24 hours after infection when the fleas were allowed to feed for 18–22 hours (high-frequency group). This suggests that EPT after 4 hours was below the detection level of our plating assay and that post-transmission bacterial amplification in the blood during hours 4–22 was required. Alternatively, since the high-frequency group fleas had constant access to blood meals during the 18–22 hour feeding period, multiple feeding attempts by individual fleas may have resulted in more CFUs transmitted. Cat fleas from the high-frequency feeding group were capable of successively transmitting *Y*. *pestis* on each of the first 3 days of infection in 2 out of 3 experiments. Not surprisingly, fleas in the high-frequency group did not transmit after the first week, because nearly all had cleared the infection by then, and in the few chronically infected fleas the infection was usually confined to the midgut.

In contrast to EPT, fleas fed at low or moderate frequency regularly transmitted *Y*. *pestis* during weeks 2–4 after infection, the time frame in which blockage and heavy proventricular infections occurred, and therefore presumably due to the proventricular biofilm-dependent regurgitative mechanism. Mass transmission was frequently recorded without identifying a blocked flea. Partial blockage may explain these transmission events, because most infected cat fleas fed at low or moderate frequency had substantial amounts of biofilm in their proventriculus (Fig 3). Incomplete blockage of the proventriculus has previously been noted to be sufficient for flea species including *Nosopsyllus fasciatus*, *O*. *montana*, and *X*. *cheopis* to reflux bacteria into a bite site [49,50]. About half of the positive transmission events in the moderate-frequency feeding group and all of the events in the high-frequency group occurred without the identification of a blocked flea. For cat fleas that fed at low frequency following infection, approximately 95% of transmission events occurred when at least one blocked flea attempted to feed. However, the diagnosis of a blocked cat flea did not always coincide with transmission, consistent with previous observations that the bites of blocked rat fleas do not always result in transmission [24,51].

As has been reported for the EPT mechanism, our results indicate that *C*. *felis* also transmits less efficiently than *X*. *cheopis* by the proventricular biofilm-dependent mechanism. In similar experimental conditions (twice-weekly maintenance feeds after a comparable infectious blood meal) >80% of *X*. *cheopis* fleas develop chronic infections and ~40% develop complete blockage [43,52,53]; compared to 43% and 12%, respectively, for *C*. *felis* (Fig 2C, Table 1). Correspondingly, the MLE transmission efficiency 14 days after infection for *X*. *cheopis*, when the incidence of blockage is highest, was 14.1% [36]. In comparison, our calculated transmission efficiency for cat fleas fed at low frequency was 7-fold lower (2.1%) on day 13. Furthermore, the numbers of CFU transmitted by *X*. *cheopis* in mass transmission experiments were >10-fold higher than we observed for *C*. *felis* [54], even without taking into account the longer feeding period (4 hours vs. 1 hour) which likely inflated CFU numbers transmitted by cat fleas due to post-transmission multiplication. Thus, in three important aspects (infection potential, proventricular blockage potential, and transmission efficiency), the vector competence of cat fleas is lower than *X*. *cheopis*, even when they are limited to a twice-weekly feeding schedule. Although feeding durations in these studies were not identical (4 hours for *C*. *felis* vs. 1 hour for *X*. *cheopis*), the data suggest that feeding frequency alone cannot completely account for the disparity in vector competence between cat fleas and *X*. *cheopis*.

Variation in foregut anatomical features and physiology, in addition to frequency and duration of feeding, may partially explain differences in vector competence between species. Disparity in esophageal and proventricular width may cause increased flow rates of blood passing through the cat flea proventriculus relative to the rat flea, making it more difficult for a biofilm to completely block the cat flea proventriculus. Consistent with this notion, the cat flea proventricular muscles are over twice as thick as those of the rat flea, and the esophagus is narrower, potentially influencing not only flow rate, but the suction strength of blood being forced in and out of the proventriculus during digestion of the blood meal. Stronger proventricular contractions and higher hydrodynamic blood flow rates between the midgut and the proventriculus may potentially dislodge adherent *Y*. *pestis* aggregates from the proventricular spines. In addition, the size and arrangement of the spines also varies between cat and rat fleas, with rat fleas tending to have longer and broader spines [55]. In combination, these traits could make chronic bacterial infection of the cat flea proventriculus less likely.

Two other anatomical differences of the cat flea foregut may also impede regurgitative transmission. We noted a thick band of muscle tissue surrounding the base of the esophagus in *C*. *felis* that was absent in *X*. *cheopis*. This may function as a sphincter that restricts backflow of proventricular contents into the esophagus (S1 Video). In *C*. *felis*, contractions originating from the musculature that surrounds the esophagus appear to force blood posteriorly from the proventriculus towards the midgut (S1A Video). In contrast, proventricular contractions of *X*. *cheopis*, which lack a muscle layer at the base of the esophagus, produce lateral but not backward-directed forces (S1B and S2 Videos). It has been noted previously that *Y*. *pestis*-induced blockage of *Xenopsylla* spp. results in distension of the esophagus as well as the proventriculus [18,50]. We also observed significant extension of the proventricular biofilm into the esophagus of blocked *X*. *cheopis* that greatly expanded the base of the esophagus. In contrast, biofilm extension into the esophagus was infrequent in cat fleas, where the biofilm was typically confined to the proventriculus and the esophagus remained narrow (Fig 8). Expansion of the esophagus could increase the total surface area of infectious biofilm coming into contact with incoming blood, increasing the probability of bacteria being dislodged and refluxed into the dermis of a host. These observations suggest that the muscle layer at the base of the cat flea esophagus tends to confine the biofilm to the proventriculus, potentially resulting in reduced transmission efficiency. Regurgitation from the midgut or proventriculus has also been proposed as the mechanism behind EPT [30,56], so these differences in foregut anatomy could also explain the relative inefficiency of EPT by cat fleas.

Cat fleas can become chronically infected with and transmit both *Bartonella* spp. and rickettsiae [57,58]. Transmission of these pathogens can occur when copious, virulent organisms within the fecal material of infected cat fleas are rubbed or scratched into the bite site [2,4,59,60]. Because *Y*. *pestis* was culturable from infected cat flea feces, we considered the possibility that fleas probing through fecal deposits could mechanically push bacteria through the membrane and contaminate the blood meal reservoir. This appeared to be unlikely, because the fleas fed from below the membrane and the vast majority of feces dropped to the bottom of the capsule or were absorbed by the gauze affixed to the membrane surface. In addition, feeding units with membranes that were defecated upon for 4 hours by recently infected cat fleas were drained of blood, the reservoir (but not the outer surface of the membrane) was washed with sterile PBS, refilled with sterile blood; and then ~100 uninfected cat fleas were allowed to feed through the soiled surface of the membrane for 4 or 24 hours. In two independent experiments, no bacteria were recovered from the blood meal reservoirs. Therefore, in our feeding system, this mode of transmission was not detectable; however, the potential for transmission via fecal contamination of a bite site under alternative feeding conditions cannot be dismissed.

*C*. *felis* is a common domestic and peridomestic flea in parts of Africa and China where plague is endemic. Since it feeds on a variety of hosts, including humans and wild rodent reservoirs of *Y*. *pestis*, it has been considered as a vector that could be responsible for transmitting *Y*. *pestis* to humans [10,12]. Our work suggests that the most likely scenario for *C*. *felis* to become infective would be after its host dies of septicemic plague. This would lead to a potential multi-day lapse in feeding, a reduction in flea body temperature, and an imperative for the flea to find a new host. Lapses in blood meal acquisition could give *Y*. *pestis* an opportunity to establish an infectious biofilm in the cat flea digestive tract. Even then, however, our results indicate that cat flea vector competence is less than that of other common fleas in these endemic areas, notably *X*. *cheopis*. In addition to feeding frequency, other factors that we were not able to assess might also affect the vector competence of *C*. *felis*, including population genetic differences, digestive tract microbiota, and host blood source. For example, our laboratory colony of *C*. *felis* has a very limited commensal microbiota, primarily consisting of intracellular endosymbionts [61]. Wild fleas may have a more varied and extensive microbiota, and it has been hypothesized that geographic and temporal differences in commensal flora among intraspecific populations could influence vector competence [62,63]. Importantly, ecological factors besides vector competence may also moderate the overall vectorial capacity of the cat flea. For example, cat fleas in a plague focus in Uganda may feed almost exclusively on humans and domestic animals, and not on small rodent reservoirs of plague [12]. In summary, feeding behavior, differences in host preference, and possibly foregut anatomy, rather than an intrinsic resistance to infection and proventricular blockage, likely limits transmission of *Y*. *pestis* by the cat flea.

## Supporting Information

S1 Video Differential proventricular contraction forces generated by *C*. *felis* and *X*. *cheopis*. Female *C*. *felis* (**A**) and *X*. *cheopis* (**B**) fleas were collected after feeding on sterile blood, placed on ice, and dissected in PBS. Rhythmic contraction of the proventricular musculature causes churning of the midgut contents, possibly to aid in digestion of the blood meal. Contractions occur about twice every second in both flea species, which is consistent with external observations of the proventricular contractions in live fleas (S2 video). In *C*. *felis*, contractions originating from the musculature that surrounds the base of the esophagus are directed backward towards the midgut. In contrast, *X*. *cheopis* proventricular contractions are lateral, with little to no movement of the tissue surrounding the esophagus. The stomodeal valve (connected to the base of the proventriculus and extending into the midgut) appears to contract coordinately with the proventricular musculature. However, the stomodeal valve did not stain for actin (Fig 2E) and is likely not predominantly composed of muscle. The stomodeal valve appears to be thicker and extends further into the midgut in *C*. *felis* than in *X*. *cheopis*. These 15-second, real-time videos were acquired using cellSens software and an Olympus DP72 camera during the first 2 minutes immediately following dissection. No cover slip was applied.
(MP4)

S2 Video Proventricular contractions of an undissected *X*. *cheopis* flea shortly after feeding. Proventricular contractions and peristalsis of the midgut observed externally in a live, recently fed female *X*. *cheopis*. The white circle indicates the location of the proventriculus. This 15-second, real-time video was acquired using cellSens software and an Olympus DP72 camera.
(MP4)
